# Supplementary material for: Epidemiology of Burkholderia pseudomallei, Streptococcus suis, Salmonella spp., Shigella spp. and Vibrio spp. infections in 111 hospitals in Thailand, 2022
Source: PLOS Glob Public Health. 2025 Mar 25;5(3):e0003995. doi: 10.1371/journal.pgph.0003995 (PMC11936208; doi:10.1371/journal.pgph.0003995)
Supplement: S4 Table — (Word) [file pgph.0003995.s004.docx]

- **S4 Table. Factors associated with the incidence of cases with culture-confirmed *Vibrio* spp. infection per 100,000 population in 74 provinces in Thailand, 2022**

| **Factors*** | **No. of cases** | **Population** | **Crude incidence rate ratio (95% CI)** | **P value** | **Adjusted incidence rate ratio (95% CI)** | **P value** |
| --- | --- | --- | --- | --- | --- | --- |
| Health regions |  |  |  |  |  |  |
| 1 | 23 | 5,725,000 | 1.1 (0.3-3.9) | 0.033 | 2.2 (0.5-9.0) | 0.032 |
| 2 | 51 | 3,723,000 | 3.5 (1.0-12.5) |  | 6.8 (1.6-28.6) |  |
| 3 | 20 | 2,996,000 | 1.5 (0.4-5.7) |  | 2.7 (0.6-11.5) |  |
| 4 | 27 | 5,275,000 | 1.0 |  | 1.0 |  |
| 5 | 147 | 5,430,000 | 6.2 (2.0-19.3) |  | 10.0 (2.7-36.7) |  |
| 6 | 112 | 6,312,000 | 5.3 (1.7-16.5) |  | 5.6 (1.6-20.0) |  |
| 7 | 52 | 5,131,000 | 2.8 (0.7-10.6) |  | 5.4 (1.2-23.6) |  |
| 8 | 91 | 5,629,000 | 3.3 (1.0-10.9) |  | 6.7 (1.7-26.3) |  |
| 9 | 93 | 6,924,000 | 4.7 (1.3-17.2) |  | 8.5 (2.0-35.6) |  |
| 10 | 64 | 4,702,000 | 3.5 (1.0-12.4) |  | 6.9 (1.6-29.2) |  |
| 11 | 57 | 4,561,000 | 4.6 (1.4-14.9) |  | 8.0 (2.1-31.0) |  |
| 12 | 72 | 5,084,000 | 3.2 (1.0-10.5) |  | 6.0 (1.6-23.1) |  |
| Gross provincial product* | - | - | 1.24 (0.96-1.59) | 0.10 | 1.16 (0.84-1.60) | 0.37 |
| Pig density* | - | - | 1.13 (0.88-1.46) | 0.34 | 0.93 (0.71-1.22) | 0.61 |
| Poultry density* | - | - | 1.11 (0.86-1.43) | 0.43 | 1.30 (0.95-1.78) | 0.10 |

- * Data were from 111 public referral hospitals in Thailand. All three continuous variables were standardized by centering at the mean values and divided by the standard deviation. The multivariable model consists of 809 patients with *Vibrio* spp. infection from 61,492,000 population over one year in 2022.
