# Supplementary material for: Epidemiology of Burkholderia pseudomallei, Streptococcus suis, Salmonella spp., Shigella spp. and Vibrio spp. infections in 111 hospitals in Thailand, 2022
Source: PLOS Glob Public Health. 2025 Mar 25;5(3):e0003995. doi: 10.1371/journal.pgph.0003995 (PMC11936208; doi:10.1371/journal.pgph.0003995)
Supplement: S5 Table — (Word) [file pgph.0003995.s005.docx]

**S5 Table.** **Total number of cases following selected notifiable bacterial diseases (NBDs) diagnosed by culture in 2022 compared with the yearly incidence between 2012-2015 in 49 hospitals where paired data were available**

| **Infections*** | **2022*** | **2012-2015**** |
| --- | --- | --- |
| *Brucella* spp. infection | 5 | 1 |
| *Burkholderia pseudomallei* infection | 2,661 | 1,651 |
| *Corynebacterium diphtheriae* infection | 6 | 10 |
| *Neisseria gonorrhoeae* infection | 13 | 18 |
| *Neisseria meningitidis* infection | 1 | 2 |
| Non-typhoidal *Salmonella* spp. | 2,214 | 3,448 |
| *Salmonella* *enterica* serovar Paratyphi infection | 5 | 8 |
| *Salmonella* *enterica* serovar Typhi infection | 6 | 34 |
| *Shigella* spp. infection | 21 | 91 |
| *Streptococcus suis* infection | 473 | 172 |
| *Vibrio* spp. infection | 438 | 567 |

Cases were defined as an inpatient with a clinical specimen culture positive for a pathogen during the evaluation period. * This study ** Data were from the reference 15.
