# Supplementary material for: Epidemiology of Burkholderia pseudomallei, Streptococcus suis, Salmonella spp., Shigella spp. and Vibrio spp. infections in 111 hospitals in Thailand, 2022
Source: PLOS Glob Public Health. 2025 Mar 25;5(3):e0003995. doi: 10.1371/journal.pgph.0003995 (PMC11936208; doi:10.1371/journal.pgph.0003995)
Supplement: S1 Text — (Word) [file pgph.0003995.s006.docx]

**S2 Text.** **Example of reporting criteria of relevant notifiable diseases in Thailand**

**Brucellosis**

Reporting suspected, probable and confirmed cases to Report506 (NSS in Thailand) using

ICD-10 codes: A23 (including A23.0, A23.1, A23.2, A23.3, A23.8 and A23.9)

- Suspected case is defined as a case with clinical criteria (at least one symptom) and a risk factor (e.g. drinking non-pasteurized milk or milk products or history of exposure to animals, animal tissues or secretions from animals)
- Probable case is defined as a case with clinical criteria, plus an epidemiological history associated with confirmed case or animals or a positive result according to presumptive diagnosis of laboratory criteria
- Confirmed case is defined as a case with clinical criteria plus a positive result according to specific diagnosis of laboratory criteria

Presumptive diagnosis of laboratory criteria includes

- Rose Bengal test (RBT) from serum is positive
- Lateral flow assay (LFA) from serum is positive

Specific diagnosis of laboratory criteria includes

- Pathogen identification based on bacterial culture or PCR
- Serology using Standard (Tube) agglutination test (SAT) using paired serum or ELISA or Coomb test from serum showing antibody level higher than that found using SAT 4-6 folds in case of acute infection and 16-256 folds in case of chronic infection

In Table 1. Food poisoning (a notifiable disease in the NSS) was compared with Non-typhoidal *Salmonella* spp. infection, and cholera (a notifiable disease in the NSS) was compared with *Vibrio* spp. infection

**Reference:**

Division of Epidemiology, Department of Disease Control. Case Definition for Communicable Diseases Surveillance, Thailand, 2020. Nonthaburi: Division of Epidemiology, Department of Control (TH); 2020. Available at <https://ddc.moph.go.th/uploads/publish/1142920210518092542.pdf>
