# Supplementary material for: Epidemiology of Burkholderia pseudomallei, Streptococcus suis, Salmonella spp., Shigella spp. and Vibrio spp. infections in 111 hospitals in Thailand, 2022
Source: PLOS Glob Public Health. 2025 Mar 25;5(3):e0003995. doi: 10.1371/journal.pgph.0003995 (PMC11936208; doi:10.1371/journal.pgph.0003995)
Supplement: S2 Text — (Word) [file pgph.0003995.s007.docx]

**S1 Text. Stata code for fitting the multivariable Poisson regression models**

* Load the data

import excel using "TH_2022_74_provinces_NBD.xls" , sheet("Sheet1") firstrow

* Fit multilevel univariable Poisson regression model

global var "GPP_2021_S Pig_density_S Poultry_density_S"

mepoisson Bps_PROV ib4.Health_region ///

, intmethod(mcaghermite) exposure(Pop_2022) ||Province_code:, irr

foreach var of global var{

mepoisson Bps_PROV `var’ ///

, intmethod(mcaghermite) exposure(Pop_2022) ||Province_code:, irr

}

* Fit multilevel multivariable Poisson regression model with province-specific random effects.

mepoisson Bps_PROV ib4.Health_region GPP_2021_S Pig_density_S ///

Poultry_density_S, intmethod(mcaghermite) exposure(Pop_2022) ///

||Province_code:, irr

* Estimate p value for Health region variable

est store a

quietly mepoisson Bps_PROV GPP_2021_S Pig_density_S ///

Poultry_density_S, intmethod(mcaghermite) exposure(Pop_2022) ///

||Province_code:, irr

est store b

lrtest a b

mepoisson Nts_PROV ib7.Health_region GPP_2021_S Pig_density_S ///

Poultry_density_S, intmethod(mcaghermite) exposure(Pop_2022) ///

||Province_code:, irr

mepoisson Ssu_PROV ib11.Health_region GPP_2021_S Pig_density_S ///

Poultry_density_S, intmethod(mcaghermite) exposure(Pop_2022) ///

||Province_code:, irr

mepoisson Vib_PROV ib4.Health_region GPP_2021_S Pig_density_S ///

Poultry_density_S, intmethod(mcaghermite) exposure(Pop_2022) ///

||Province_code:, irr
