# Supplementary figures and images for: Epidemiology of Burkholderia pseudomallei, Streptococcus suis, Salmonella spp., Shigella spp. and Vibrio spp. infections in 111 hospitals in Thailand, 2022
Source: PLOS Glob Public Health. 2025 Mar 25;5(3):e0003995. doi: 10.1371/journal.pgph.0003995 (PMC11936208; doi:10.1371/journal.pgph.0003995)

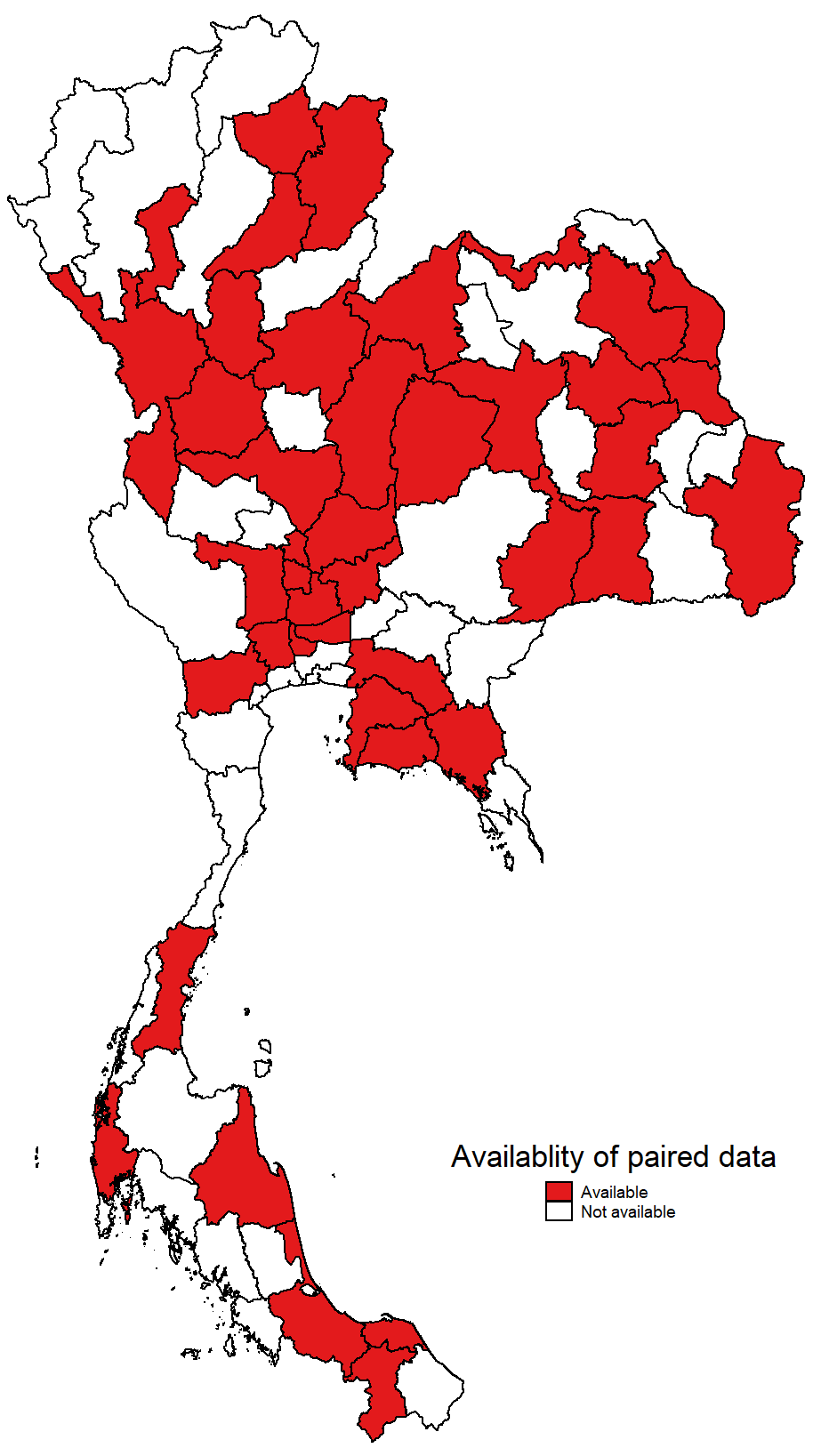

Supplement: S1 Fig — Map created by the authors using STATA version 14.2 (StataCorp, College Station, TX) and a base layer map from GADM (https://gadm.org/download_country.html) (term of use: https://gadm.org/license.html) (Image) [file pgph.0003995.s008.tif]
