# Supplementary material for: Epidemiology of Burkholderia pseudomallei, Streptococcus suis, Salmonella spp., Shigella spp. and Vibrio spp. infections in 111 hospitals in Thailand, 2022
Source: PLOS Glob Public Health. 2025 Mar 25;5(3):e0003995. doi: 10.1371/journal.pgph.0003995 (PMC11936208; doi:10.1371/journal.pgph.0003995)
Supplement: Attachment — Submitted filename: response_to_reviewers 2011 11 03.docx [file pgph.0003995.s009.docx]

Re: PLOS Global Public Health Decision: Revision required [PGPH-D-24-02039] - [EMID:0374f8e8e7205a8c]

Dear Editor,

Thank you for your e-mail concerning the above manuscript. We are grateful for the helpful comments made by the reviewers, and have addressed them as detailed below.

Journal Requirements:
1. Please amend your detailed Financial Disclosure statement. This is published with the article. It must therefore be completed in full sentences and contain the exact wording you wish to be published.

**Please only choose the relevant sentences from below**

1) Please clarify all sources of funding (financial or material support) for your study. List the grants (with grant number) or organizations (with url) that supported your study, including funding received from your institution. 
2) State the initials, alongside each funding source, of each author to receive each grant.
3) State what role the funders took in the study. If the funders had no role in your study, please state: “The funders had no role in study design, data collection and analysis, decision to publish, or preparation of the manuscript.”
4) If any authors received a salary from any of your funders, please state which authors and which funders.

*Answer: Revised as suggested.*

2. We have noticed that you have uploaded Supporting Information files, but you have not included a list of legends. Please add a full list of legends for your Supporting Information files after the references list.

*Answer: Revised as suggested.*

3. Figures 1 and 2: please (a) provide a direct link to the base layer of the map (i.e., the country or region border shape) and ensure this is also included in the figure legend; and (b) provide a link to the terms of use / license information for the base layer image or shapefile. We cannot publish proprietary or copyrighted maps (e.g. Google Maps, Mapquest) and the terms of use for your map base layer must be compatible with our CC-BY 4.0 license.

Note: if you created the map in a software program like R or ArcGIS, please locate and indicate the source of the basemap shapefile onto which data has been plotted.

If your map was obtained from a copyrighted source please amend the figure so that the base map used is from an openly available source. Alternatively, please provide explicit written permission from the copyright holder granting you the right to publish the material under our CC-BY 4.0 license.

Please note that the following CC BY licenses are compatible with PLOS license: CC BY 4.0, CC BY 2.0 and CC BY 3.0, meanwhile such licenses as CC BY-ND 3.0 and others are not compatible due to additional restrictions.

If you are unsure whether you can use a map or not, please do reach out and we will be able to help you. The following websites are good examples of where you can source open access or public domain maps: 
* U.S. Geological Survey (USGS) - All maps are in the public domain. ([http://www.usgs.gov](https://url.uk.m.mimecastprotect.com/s/s7KACgxYvHAGy43cohAF4fs4l?domain=usgs.gov)) 
* PlaniGlobe - All maps are published under a Creative Commons license so please cite “PlaniGlobe, [http://www.planiglobe.com](https://url.uk.m.mimecastprotect.com/s/qD07CjvQyfnRmZrH5iWFmr0tu?domain=planiglobe.com), CC BY 2.0” in the image credit after the caption. ([http://www.planiglobe.com/?lang=enl](https://url.uk.m.mimecastprotect.com/s/vxscCkwQzCO5BoEc9s9FGTrI8?domain=planiglobe.com)) 
* Natural Earth - All maps are public domain. ([http://www.naturalearthdata.com/about/terms-of-use/](https://url.uk.m.mimecastprotect.com/s/xVRzClvQAf2XE6rU1tZFzcJuW?domain=naturalearthdata.com))

*Answer: The following sentences have been added in the legend of the Fig 1 and 2, “Map created by the authors using STATA version 14.2 (StataCorp, College Station, TX) and a base layer map from GADM (*[*https://gadm.org/download_country.html*](https://gadm.org/download_country.html)*) (term of use:* [*https://gadm.org/license.html*](https://gadm.org/license.html)*)”*

**Reviewer #1**

Overall comments
Jitpeera et al. analyse the incidence of 11 different bacterial pathogens from data they have collected from the majority of Thailand’s hospitals in 2022, and then compare incidence from some of these regions with paired data from 2012-2015. In-hospital mortality and associations with local agricultural animal density, economic data and the health region are also investigated. The scale of the data and observed trends are valuable to the field and overall the data is well presented and described, and conclusions are appropriately supported by the data. However, several points need to be clarified and some recommendations for additional data presentation are included below. While the discussion raises good points, this section would benefit from expanding slightly to put more details into context, particularly for readers not familiar with these bacterial pathogens. Unable to comment on the validity of the statistical analysis.

Major comments
1. Line 47. At some point in the manuscript (methods?) the authors should explain why there is no data from Bangkok.

*Answer: The following sentences have been added to the method section for clarity, “Public referral hospitals in Bangkok (health region 13) were managed under Department of Medical Services, MoPH (i.e. not under the Health Administration Division, MoPH). Therefore, Bangkok was not included in the study.”*

2. Line 58. “The incident rate of NTS infection was not associated with geographical region.” This sentence is not accurate as written, and should be rephrased to make it clear that NTS incidence does vary by region, perhaps by as much as 10-20 times (from the data shown in Fig 2F). I believe the authors meant to say that NTS incidence was generally high across all regions of Thailand, and there is no clear epicentre of infection.

*Answer: The sentence has been revised as suggested, “The incidence rate of NTS infection was generally high across all geographical regions.”*

3. Line 59 – 61. The authors highlight that the incidence fecal-oral transmitted NBDs was lower and that of B. pseudomallei and S. suis was higher. The route of transmission of the later should be mentioned (perhaps in the discussion).

*Answer: The following sentences have been added to the discussion section as suggested, “Melioidosis is usually acquired by skin inoculation, ingestion or inhalation of B. pseudomallei in soil and water. Preventive measures include wearing appropriate protective clothing and footwear when contacting with soil or water, consuming bottled or boiled water, and ensuring that the domestic water supply is not contaminated with B. pseudomallei.” and “Risk factors for S. suis infection include consuming undercooked pork contaminated with S. suis and exposure to pigs or pork.”*

4. Line 78-81. It should be made clear whether any of the 13 dangerous communicable diseases are bacterial and if so what they are.

*Answer: The following sentence has been added to the introduction section as suggested, “Of 13 dangerous communicable diseases, 11 were viral infections (e.g. Ebola infection) and two were bacterial infections (Yersinia pestis infection and extensively drug-resistant tuberculosis).”*

5. Line 84: It should be noted whether the ICD-10 is reliable for any of the 11 bacterial diseases described in the manuscript. The following sentence stating that microbiology lab data is not used would suggest that they are not, but this should be made clear.

*Answer: The following sentence has been added to the introduction section as suggested, “The ICD-10 is unreliable for notifiable bacterial diseases (NBDs).”*

6. Line 119-121 & Line 139 - 142: Some more specifics on how each bacterial disease was cultured and identified is warranted. Ref 23 handbook link is broken, but is dated as 2012; has there been any update to procedures/methods since? It should be made clear whether the methods for collecting data differ between the earlier timepoint and the later. This is particularly important for Burholderia and S. suis which have seen large increases in incidence over the period.

*Answer: Ref 23 has been changed to*

*“23. Department of Medical Science,* *Ministry of Public Health, Thailand. Laboratory manual for emerging diseases for regional and general hospitals. 2021. Available from:* [*https://www3.dmsc.moph.go.th/download/files/dmsc_recurrence.pdf*](https://www3.dmsc.moph.go.th/download/files/dmsc_recurrence.pdf)*”*

*The following sentence have been added to the discussion section for clarity, “Fourth, the method for collecting data between 2012 and 2015 was similar to those used in 2022, utilizing microbiology and hospital admission data and AMASS.”*

*Additional sentences have been added into discussion to take account of the possibility of improved diagnostic capacity over time.*

7. While culture is the gold standard for the diagnosis of many of these pathogens, the authors should note in the discussion if there are any limitations with using solely culture as a readout for specific pathogens that may bias the data. This is very briefly mentioned in the discussion at line 344-346, but more detail should be included in terms of how cultured methods may vary between regions if know.

*Answer: The following sentence have been added to the discussion section for clarity, “Third, our approach focused on bacterial culture results. Therefore, the findings could be influenced by diagnostic stewardship and, capability and expertise of the microbiology laboratories. Methods of bacterial culture and identification may differ by region and over time. For example, some hospitals might have new bacterial identification automated systems in 2022, reducing the possibility of misidentification of S. suis (as Streptococcus spp.) and B. pseudomallei (as contaminants or Pseudomonas spp.).”*

8. Were any patients culture positive for more than one pathogen? Regardless of answer this should be stated if known.

*Answer: The following sentence has been added to the discussion section for clarity, “Fifth, the AMASS did not generate reports for patients who were culture positive for more than one pathogen. Such patients would be counted separately for each pathogen.”*

9. Line 146-147: How many patients had multiple admissions? Explain why mortality data was based only on the first admission.

*Answer: The following sentences have been added to the discussion and method sections for clarity, “Sixth, the AMASS did not generate reports for number of admissions among patients who were culture positive for pathogens of interest.” and “This approach was taken to avoid overestimating mortality due to other causes (e.g. healthcare-associated infections and road accidents) that might be associated with the subsequent admissions. Some patients with certain NBDs (e.g. B. pseudomallei infection) may need several months of treatment to achieve culture negativity.”*

10. Line 165-166. It should be noted what biases the paired data introduce in the analysis. Eg. what regions/ hospital types were less/more represented in the 2012-2015 dataset relative to that of the 2022 dataset.

*Answer: The following sentences have been added to the result sections for clarity, “The 46 hospitals were located in all 12 health regions. There was no difference in proportions of health regions having and not having paired data (p=0.31). Nineteen hospitals were Level A (41%), 20 Level S (44%) and 7 Level M1 (15%). There was borderline evidence showing that the proportion of Level M1 among hospitals with paired data was lower than those without paired-data (15% [7/46] vs. 31% [20/65], p=0.079).” Figure S1 has also been added for clarity.*

11. Line 196: Which regions are included in the 2012-2015 dataset? Can this be displayed on a map in a figure?

*Answer: All regions are included in the 2012-2015 dataset. The additional information has been added as described above. Figure S1 has also been added for clarity.*

12. Line 288: This paragraph compares the collected data with that of another dataset from the national surveillance system, and concludes that there are significant differences between the two datasets in terms of incidence and mortality. The argument is made in the discussion in lines 305 -306 that “local data can supplement and monitor the performance of the national surveillance systems”. However, it is not explained why such a disparity exists between the two datasets, and it is not obvious to the reader why; this needs to be clearly explained in the discussion.

*Answer: The following sentences have been added to the discussion section, “Underreporting cases and deaths following B. pseudomallei, S. suis and other infections to the national surveillance system is consistent with the previous finding [11,16]. It is likely that many healthcare workers responsible for reporting to the national surveillance system are still unaware that all cases and deaths following culture-positive NBDs must be reported [16] and that ICD-10 of those culture-confirmed cases in the hospitals’ health information systems were largely inaccurate [13,14]. Additionally, their perceptions of their workload and the belief that reporting cases is not used for outbreak response may contribute to underreporting [28]. Further studies on underreporting and on automating reporting systems utilizing laboratory data from healthcare facilities are required.”*

13. Line 320: specify “increase from 2012-2015 to 2022”. The authors should note, as they have done for burkholderia, whether the increase observed for S. suis could have been caused by changes in diagnostic procedures/methods etc.

*Answer: The additional discussion has been added as described above.*

14. Line 327: specify the “fecal-oral transmitted diseases”. Transmission of burkholderia and S. suis should be discussed as a point of difference.

*Answer: Routes of transmission for B. pseudomallei and S. suis infections have been added to the discussion section as described above.*

15. Line 328-330: This sentence is very general. Be specific about what interventions could decrease incidence and why.

*Answer: The following sentences have been added as suggested, “The DDC will utilize the data to additionally implement and enforce behavioral interventions, education and food biosafety in the country [38]. These interventions include educating at-risk populations, such as pig farmers, and increasing public disease awareness and prevention efforts through public health officers and village health volunteers. Additionally, food biosafety measures will be enforced in collaboration with relevant stakeholders, emphasizing proper handling and processing of pork at abattoir, market and consumer levels to prevent contamination and transmission.”*

16. Line 351: In the concluding paragraph only melioidosis and S. suis are mentioned as requiring specific public health inventions. While it is true that these have increased dramatically and have the highest associated mortality, the data shows that NTS and cholera, despite decreasing over the same period, continue to have high incidence in Thailand and high mortality. Therefore NTS and cholera should be highlighted as continuing to have high burden in the country and requiring specific public health interventions.

*Answer: The conclusion paragraph has been revised as suggested, “In conclusion, the burden of melioidosis and S. suis infections is increasing in Thailand. Their incidence rates are higher in some regions than in others. Although the incidence of NTS infection and vibriosis are decreasing, they are still associated with a high number of cases and deaths. Specific public health interventions to reduce the burden of melioidosis, S. suis infection, NTS infection and vibriosis are urgently required.”*

17. The comparison is between a multiyear timeframe (2012-2015) and a single year. Was there anything different or special about the 2022 year (eg. climate) that may have lead to a changes in disease transmission? Have any vaccination programs/antimicrobial treatment/general healthcare programs for any of these pathogens been introduced since 2015 which may have affected disease incidence?

*Answer: The evidence related to climate change is still limited. We agree that the improvement of bacterial identification and healthcare systems has partially contributed to some part of the change, as expanded in the discussion section. The COVID-19 situation in 2022 was much better compared to 2020 and 2021. We unofficially observed that the total number of patient admissions in each hospital was returning to pre-COVID-19 levels (2019). Nonetheless, the bacterial identification in Thailand between 2012-2015 was relatively good. Therefore, we consider and discuss that the spread of S. suis contaminated pork products is likely the main cause. The following sentences have been added for clarity, “The increased incidence rate of S. suis infection could partially be associated with improvements in diagnostic stewardship and bacterial identification in public hospitals in Thailand over time. Nonetheless, these factors alone cannot explain the nearly three-fold increase in S. suis infection in the country.”*

18. Is there patient meta data available that could help explain changes in incidences and affected populations eg. age, gender, comorbidities, coinfections with more than one NBD?

*Answer: We do not have the patient meta data. The following sentence is in the discussion section for clarity, “Fourth, our approach utilizes summary data and could not evaluate individual-level clinical data and microbiology laboratory data in details.”*

Figures/Tables:
1. Figure 2:
a. The colours at the high end of incidence are similar and hard to differentiate. Recommend adapting the colours (perhaps to a 2 or 3 colour system?) to make the difference incidence levels easier to distinguish.b. The size of the numbers in the colour legend should be increased to a more legible level.
c. Fig legend should note the pathogen for each of the numbered graphs.

*Answer: The figures have been revised as suggested.*

2. The authors compare paired data from 2022 with that of 2012-2015. It should be made clear what regions/provinces are included in the this temporal comparison. Perhaps a map highlighting the regions/provinces incorporated? A geographical figure similar to Fig 2 that shows the % or fold change in incidence of each NBD during this period may work. Or could be a column in a data table.

*Answer: The Figure S1 has been added as suggested.*

3. Recommend that the authors include a table summarising all data for easy comparison between pathogens eg. total patients, total deaths, % deaths, % change from 2012-2015 data.

*Answer: The Table S5 has been added as suggested.*

Minor comments
1. Line 135: Should be 11 pathogens, not 12.

*Answer: We confirmed that there are 11 pathogens*

2. Ref 23 url does not open.

*Answer: The new reference has been used.*

3. Line 201: “the time periods” should be specified.

*Answer: Revised as suggested.*

4. Line 225: “none died” should be rephrased to “no associated mortality” or similar.

*Answer: The following sentence has been revised as suggested, “no deaths following the infection were observed”*

5. Line 289-290. Make clear that the second figures in the brackets are from national surveillance and quantify the difference eg. in fold or %

*Answer: The following sentences have been revised as suggested, “In 2022, the total number of cases with B. pseudomallei infection diagnosed by culture (4,407 cases) was higher than that reported to the national surveillance systems (3,357 cases, a 1.3-fold difference, Table 1). The total number of deaths following B. pseudomallei infection diagnosed by culture (1,129 deaths) was higher than that reported to the national surveillance systems (157 deaths, a 7.2-fold difference). The total number of cases with S. suis infection diagnosed by culture (867 cases) was also higher than those reported to the national surveillance systems (383 cases, a 2.3-fold difference). The total number of deaths following S. suis infection diagnosed by culture (134 deaths) was also higher than that reported to the national surveillance systems (10 deaths, a 13.4-fold difference).”*

6. Line 300-301: Make clear that increase is relative to 2012-2015 data.

*Answer: The sentence has been revised as suggested, “Our findings provide evidence that the incidences of melioidosis and S. suis infections in Thailand in 2022 are increasing compared to the data from 2012-2015 and associated with a high number of deaths.”*

7. Line 310. Sentence not grammatically correct, rephrase.

*Answer: The sentence has been revised as follows, “The finding that there were more than 1,200 deaths following melioidosis in 2022 is alarming.”*

8. Line 316-317: “spurious” is not the right word here.

*Answer: The sentence has been revised as follows, “The association between poultry density and melioidosis has never been observed, and could be due to a collinearity with residual confounding factors (e.g. agricultural occupation).”*

9. Line 332-33: Quantify the % of referral hospitals in the country included in the data.

***Answer: The sentences have been added for clarity as follows, “******According to data from the Healthcare Accreditation Institute Thailand [47], the public referral hospitals included in this study represented 56.6% (120/212) of hospitals with at least 180 beds, 66.4% (81/122) of hospitals with at least 300 beds and 70% (42/60) of hospitals with at least 500 beds in Thailand.” The reference has been replaced with***

***“47.*** ***Healthcare Accreditation Institute, Thailand. Resource data: general data of hospitals. 2022. Available from: https://data.go.th/dataset/hospital”***

**Reviewer #2**

The study presents a thorough analysis of notifiable bacterial diseases (NBDs) in Thailand, providing valuable insights into emerging epidemiological trends. By leveraging comprehensive microbiology and hospital admission data from a large network of public referral hospitals, the authors address a significant knowledge gap regarding the incidence and distribution of NBDs in low- and middle-income countries. The use of multivariable Poisson random-effects regression models and the comparison with historical data (2012-2015) strengthen the robustness of the findings. However, there are some major and minor comments and areas that warrant further consideration for clarification and improvement.

**Major comments**

1. Please summarize the study that was performed in 2012-2015 as this is used as comparator. Why was this period chosen as a comperator? Please explain why chosen for these eleven bacterial diseases. And where these already notifiable pathogens in 2012 for example?

*Answer: The following sentences have been added for clarity, “The previous study [15] and the period between 2012-2015 were chosen as the comparator because it was the only study that collected both microbiology and hospital admission data and used the same analytical approach for the 11 NBDs.” The 11 diseases being evaluated have been classified as NBDs in Thailand since 2002, with melioidosis being the most recent addition that year.*

At the moment, I do not understand what you are comparing your results to and how to interpretate the major increases and decreases in incidence levels. For example, if the regions where melioidosis is known to be highly endemic were not included in the 2012-2015 study, but are now included in the 2022 study due to its larger scope, this obviously explains the increase in incidence. Without this information (or adjustment for this fact) in the analysis, it’s not possible to interpretate the incidence increases or decreases as mentioned below under a to f. It may be true that there’s an increase in awareness and subsequent identification, but then my main question remains: is the incidence increasing or is the detection increasing? The authors should be more critical about this distinction.

*Answer: The comparison between NBDs in 2022 and those between 2012-2015 was performed only in hospitals where paired data were available. Therefore, the regions (hospitals) where melioidosis is known to be highly endemic will only be included in this comparison only when the regions (hospitals) had data of both in 2022 and between 2012-2015. We also added additional information of regions that we had paired data as follows, “The 46 hospitals were located in all 12 health regions. There was no difference in proportions of health regions having and not having paired data (p=0.31). Nineteen hospitals were Level A (41%), 20 Level S (44%) and 7 level M1 (15%). There was borderline evidence showing that the proportion of Level M1 hospitals among hospitals with paired data was lower than those without paired-data (15% [7/46] vs. 31% [20/65], p=0.079).” Table S5 and Figure S1 have been added for clarity.*

a. Line 218. The authors state that the incidence of B. pseudomallei increased by 58% in 2022 compared to 2012-2015. Can the authors explain this major increase? The reasons mentioned in the discussion might be true (line 318-320), but please incorporate the suggestions above.

*Answer: The following sentence has been added into the discussion for clarity, “The observed increase was not due to the differences in the geographical regions evaluated in 2022 compared to 2012-2015, as we evaluated only provinces with paired data.”*

b. Line 242. The authors state that the incidence of NTS decreased by 37% in 2022 compared to 2012-2015. Can the authors explain this major decrease? Please elaborate on this decrease in the discussion.
c. Line 254. The authors state that the incidence of Salmonella enterica serovar Typhi decreased by 83% in 2022 compared to 2012-2015. Can the authors explain this major decrease? Please elaborate on this decrease in the discussion.
d. Line 262. The authors state that the incidence of Shigella decreased by 78% in 2022 compared to 2012-2015. Can the authors explain this major decrease? Please elaborate on this decrease in the discussion.

*Answer: For NTS, Salmonella entrica serovar Typhi, Shigella and Vibrio (fecal-oral transmitted diseases), the discussion about their decreases have been revised and expanded as follows, “The striking decrease of Salmonella enterica serovar Typhi and Shigella spp. infections (83% and 78% decrease, respectively) could be due to improvement in clean water supply, sanitation, regulatory environment, food safety, and related health intervention programs over time [41,42]. Improvement in health-seeking behaviour and access to healthcare under the universal coverage have likely contributed to the decrease [43,44]. Although the incidence rate of NTS and Vibrio spp. infection minimally decreased (37% and 25% decrease), the burden of NTS and Vibrio spp. infections is still high across the regions in 2022. The higher burden of NTS infection that those caused by typhoid is consistent with those observed in other LMICs [45]. Persistence of virulent or drug-resistant strains among susceptible population including children and people with co-morbidities may be associated with the slow reduction of the NTS [45] and Vibrio spp. infections [46]. The MoPH and related stakeholders should maintain and strengthen the public health interventions to decrease incidences of these infections further. Further studies and measures to reduce the burden of NTS infection are also required.”*

e. Line 275. The authors state that the incidence of S. suis increased by 172% in 2022 compared to 2012-2015. Can the authors explain this major increase? The reasons mentioned in the discussion might be true (line 324-328), but please incorporate the suggestions above.

*Answer: The following sentences have been added for clarity, “The increased incidence rate of S. suis infection could partially be associated with improvements in diagnostic stewardship and bacterial identification in public hospitals in Thailand over time. Nonetheless, these factors alone cannot explain the nearly three-fold increase in S. suis infection in the country.”*

f. Line 288. The authors state that the incidence of Vibrio decreased by 25% in 2022 compared to 2012-2015. Can the authors explain this major decrease? Please elaborate on this decrease in the discussion.

*Answer: as described above.*

2. Is the use of **multivariable Poisson random-effects regression models** appropriate for this type of data and analysis? Please explain why this model was chosen.

*Answer: Yes, it is appropriate for this type of data and analysis. Following sentences have been added in the method section for clarity, “Our summary data from each hospital could be considered nested within its each province. The Poisson random-effects models are appropriate for the count outcomes and allow the analysis to partition the total variation in the outcome into between-cluster variation and between-individual variation [24]. The magnitude of the effect of clustering provides a measure of the general contextual effect.”*

3. The study relies on hospital discharge summaries to define mortality. How confident are you in the reliability and consistency of these summaries across different hospitals and regions? Given the possibility of discrepancies in reporting.

*Answer: We are confident that admission data (admission date, discharge date and hospital number) and in-hospital mortality data (whether the patient died or not in the hospital) are correct because the data are used together with ICD-10 data for Diagnosis Related Groups (DRG) based hospital payments nationwide. All public referral hospitals have hospital information systems to manage hospital admission data to reimburse hospital payments. However, we do not have confident in ICD-10 data as described in the introduction section. Following sentences have been added in the method section for clarity, “Second, hospital admission data used in the analysis (including hospital number, admission date, discharge date and in-hospital mortality) were reliable, as these data were recorded electronically and required for retrospective hospital payment nationwide [45,46].”*

4. Why was Bangkok excluded from the analysis?

*Answer: The following sentences have been added in the method section for clarity, “Public referral hospitals in Bangkok (health region 13) were managed under Department of Medical Services, MoPH (i.e. not under the Health Administration Division, MoPH). Therefore, Bangkok was not included in the study.”*

**Minor comments**

1. Line 188. The authors state that 91% used the AMASS to acquire data, but how was the other 9% of the data obtained? Please elaborate.

*Answer: The other 11 hospitals (9%) had not submitted the summary AMR and NBD data to the MoPH during the study period. We did not design the study to systematically evaluate the causes of unsuccessful submission. Those could include not attending the workshops, failure to export data from either the hospital information system or the laboratory information system, lack of microbiology laboratory data in electronic format, and lack of time to update data dictionary of their own hospital to match with their own data, etc.*

2. Table 1. Suggestion to place the total number of cases and deaths in columns next to each other. This is easier for the reader to comprehend as it immediately reads as a mortality rate. Also, suggest to use the same nomenclature for the disease/causative agent. So, column 1 and column 3 should be combined to a single column. If there are discrepancies between the definition used in the AMASS and NSS, state so in the methods or supplemental files.

*Answer: Revised as suggested.*

3. Line 331-332. The authors mention that measures were taken over time that resulted in a decrease in fecal-oral transmitted diseases. The information is very brief.

The decrease in fecal-oral transmitted diseases such as typhoid and shigellosis is attributed to improvements in sanitation and clean water supply. Could other factors, such as changes in healthcare-seeking behavior, vaccination campaigns, or environmental factors, play a role in this decline? How did you isolate the specific impact of water and sanitation improvements from these other potential influences?  Please give more information on the context of the interventions. Readers should not have to go back and forth to other publications to identify this information, especially since it is considered critical information by the authors as it explains decreases in incidence levels.

*Answer: The following sentences have been revised for clarity as suggested, “The striking decrease of Salmonella enterica serovar Typhi and Shigella spp. infections (83% and 78% decrease, respectively) could be due to improvement in clean water supply, sanitation, regulatory environment, food safety, and related health intervention programs over time [41,42]. Improvement in health-seeking behaviour and access to healthcare under the universal coverage have likely contributed to the decrease [43,44]. Although the incidence rate of NTS and Vibrio spp. infection minimally decreased (37% and 25% decrease), the burden of NTS and Vibrio spp. infections is still high across the regions in 2022. The higher burden of NTS infection that those caused by typhoid is consistent with those observed in other LMICs [45]. Persistence of virulent or drug-resistant strains among susceptible population including children and people with co-morbidities may be associated with the slow reduction of the NTS [45] and Vibrio spp. infections [46]. The MoPH and related stakeholders should maintain and strengthen the public health interventions to decrease incidences of these infections further. Further studies and measures to reduce the burden of NTS infection are also required.”* *and “There are currently no licensed vaccines for melioidosis, S. suis infection and shigellosis. Vaccination for salmonella and vibriosis are not widely available and are seldom used in Thailand.”*

4. The study highlights regional differences in the incidence of B. pseudomallei and S. suis infections. Could these regional differences be influenced by varying healthcare access, diagnostic capacity, or reporting practices across provinces? How did the study address such potential confounding factors to ensure that the differences are truly epidemiological rather than artefacts of the healthcare system?

*Answer: The following sentence has been added for clarity, “The difference in the incidence by regions in Thailand is consistent with previous studies [29].” A number of clinical, animal and environmental studies showing that melioidosis is more prevalent and B. pseudomallei is more prevalent in the soil and water in those regions. The incidence is also associated with the occupation (particularly rice farmers) and environmental exposure in those regions.*

*Answer: The following sentence have been added to the discussion section for clarity, “Third, our approach focused on bacterial culture results. Therefore, the findings could be influenced by diagnostic stewardship and, capability and expertise of the microbiology laboratories. Methods of bacterial culture and identification may differ by region and over time. For example, some hospitals might have new bacterial identification automated systems in 2022, reducing the possibility of misidentification of S. suis (as Streptococcus spp.) and B. pseudomallei (as contaminants or Pseudomonas spp.).”*

*Our ongoing study in public referral hospitals is showing the similarity in diagnostic capacity between health regions but the difference in diagnostic capacity between hospital size and type (manuscript in preparation).*

5. The study calls for public health interventions to address the increasing burden of melioidosis and S. suis infections. Given the complexity of these diseases, what specific interventions would be both effective and feasible in Thailand's current public health landscape? How would you prioritize resource allocation, and what are the potential barriers to implementing these interventions across diverse regions?

*Answer: The following sentences have been added for clarity, “Melioidosis is usually acquired by skin inoculation, ingestion or inhalation of B. pseudomallei in soil and water. Preventive measures include wearing appropriate protective clothing and footwear when contacting with soil or water, consuming bottled or boiled water, and ensuring that the domestic water supply is not contaminated with B. pseudomallei [30-32].” and (for S. suis infection) “These interventions include educating at-risk populations, such as pig farmers, and increasing public disease awareness and prevention efforts through public health officers and village health volunteers. Additionally, food biosafety measures will be enforced in collaboration with relevant stakeholders, emphasizing proper handling and processing of pork at abattoir, market and consumer levels to prevent contamination and transmission.”*

While the AMASS program is scalable and user-friendly, how generalizable is the study’s approach to other low- and middle-income countries (LMICs) with different healthcare infrastructures, disease profiles, and data management capabilities? Could the reliance on computerized microbiology and hospital data present a challenge for broader adoption in less digitized health systems?

*Answer: The AMASS has been designed to work with various data systems, as long as they can export data in CSV or Excel formats. AMASS does not utilize disease profile data because it aims to use robust microbiology data (i.e., culture positivity) rather than disease profiles. Data management remains a significant barrier in LMICs, where data is often recorded only on paper, stored in Excel files, or has mixed date formats (DD-MM-YY and MM-DD-YY) in the same sheet (stored as text), etc. After implementation in Thailand, we identified these problems in few hospitals, and the MoPH will focus on and support IT improvements in microbiology laboratories across the country (personal experience; we did not systematically evaluated those barriers in this study). We believe that other LMICs, which are beginning to store data electronically, should start utilizing their data more effectively. While some hospitals may be able to use the data using the AMASS, others may encounter barriers. This could be the starting point for improving IT systems and basic IT skills among healthcare workers in these countries. Over time, we expect more hospitals in LMICs to adopt electronic systems, and AMASS can serve as a generalized tool to facilitate the data utilization and IT system improvement.*

*All contributing authors have reviewed and concurred with the revised manuscript.*

*Yours,*

*Direk Limmathurotsakul*
