# Supplementary material for: Epidemiology of Burkholderia pseudomallei, Streptococcus suis, Salmonella spp., Shigella spp. and Vibrio spp. infections in 111 hospitals in Thailand, 2022
Source: PLOS Glob Public Health. 2025 Mar 25;5(3):e0003995. doi: 10.1371/journal.pgph.0003995 (PMC11936208; doi:10.1371/journal.pgph.0003995)
Supplement: Attachment — Submitted filename: response_to_reviewers 2025 01 06.docx [file pgph.0003995.s010.docx]

*Re: PLOS Global Public Health Decision: Revision required [PGPH-D-24-02039R1] - [EMID:946d0b7bcfaebab3]*

*Dear Editor,*

*Thank you for your e-mail concerning the above manuscript. We are grateful for the helpful comments made by the reviewers, and have addressed them as detailed below.*

Journal Requirements:

Please review your reference list to ensure that it is complete and correct. If you have cited papers that have been retracted, please include the rationale for doing so in the manuscript text, or remove these references and replace them with relevant current references. Any changes to the reference list should be mentioned in the rebuttal letter that accompanies your revised manuscript. If you need to cite a retracted article, indicate the article’s retracted status in the References list and also include a citation and full reference for the retraction notice.

*Answer. Revised as suggested.*

Many thanks for addressing the major review comments from the editors. A few very minor edits still need to be made:

-line 80 - suggest replacing "information" with data

-line 86 - suggest replacing "tuberculosis" with "Mycobacterium tuberculosis" for consistency

*Answer. Revised as suggested.*

-line 90-91 - point about notifiable bacterial diseases warrants additional explanation

*Answer. The following sentence has been revised for clarity, “The ICD-10 is unreliable for notifiable bacterial diseases (NBDs) because many disease diagnoses recorded by physicians are incomplete, ICD-10 coders enter codes based solely on the diagnoses recorded by physicians, and they may tend to code only diagnoses that result in reimbursement [13,14].”*

-lines 130-2 - thanks for addressing the question about why Bangkok wasn't included, but some additional explanation for why would be welcome (e.g. data could not be accessed, and/or data are captured differently and therefore aren't comparable, etc.)

*Answer. The following sentence has been revised for clarity, “Therefore, public referral hospitals in Bangkok were not included, and their summary data were neither generated nor accessed in the study.”*

-line 189 - what is "the same analytical approach"? some additional specificity would be helpful

*Answer. The following sentence has been revised for clarity, “Specifically, AMASS was also previously used to merge both data files, deduplicate the data, and report total number of inpatients with a clinical specimen culture positive for a pathogen during the evaluation period [15].”*

-line 353 - "underreporting *of* cases and deaths..."

*Answer. Revised as suggested.*

-line 402 - decrease in... numbers? incidence?...of Salmonella enterica serovar Typhi and Shigella spp. infections

*Answer. The following sentence has been revised for clarity, “The striking decrease in incidence rates of…”*

-line 403 - decreases*

-line 407 - rates*

-lines 408-410 - what is meant by "burden" - incidence? kind of an imprecise term, could say that these pathogens cause substantial public health problems, or similar

*Answer. The following sentence has been revised for clarity, “Although the incidence rates of NTS and Vibrio spp. infection minimally decrease (37% and 25% decrease), these pathogens still cause substantial public health problems across the regions.”*

-line 441 - methods *used for*...

-line 442 - some hospitals might have *implemented* ...?

-line 452 - "Although the incidence *rates* of..."

*Answer. Revised as suggested.*

[Note: HTML markup is below. Please do not edit.]

Reviewers' comments:

Reviewer #1: Minor: The new ref 23 link is broken

*Answer. We tested the link, and confirmed the link is not broken. We suspected that data security in certain places and countries may prevent access to the website. Therefore, we have updated our data repository for this manuscript, and provided cache files from four references (Refs 12, 21, 22, 23, 35, 40 and 49) at* [*https://doi.org/10.6084/m9.figshare.25907494*](https://doi.org/10.6084/m9.figshare.25907494) *Please note that the Refs 25, 26 and 27 are not available in file formats.*

*All contributing authors have reviewed and concurred with the revised manuscript.*

*Yours,*

*Direk Limmathurotsakul*
